# FAM83D promotes ovarian cancer progression and its potential application in diagnosis of invasive ovarian cancer

**DOI:** 10.1111/jcmm.14360

**Published:** 2019-04-30

**Authors:** Qingyu Zhang, Shan Yu, Samson Ian Sam Lok, Alice Sze Tsai Wong, Yufei Jiao, Leo Tsz On Lee

**Affiliations:** ^1^ Centre of Reproduction, Development and Aging, Faculty of Health Sciences University of Macau Macau China; ^2^ School of Biological Sciences The University of Hong Kong Hong Kong China; ^3^ Department of Pathology The Second Affiliated Hospital of Harbin Medical University Harbin China

**Keywords:** FAM83D, invasive epithelial ovarian cancer, low malignant potential ovarian tumour, molecular diagnosis

## Abstract

Although invasive epithelial ovarian cancer (IOC) and low malignant potential ovarian tumour (LMP) are similar, they are associated with different outcomes and treatment strategies. The current accuracy in distinguishing these diseases is unsatisfactory, leading to delays or unnecessary treatments. We compared the molecular signature of IOC and LMP cases by analysing their transcriptomic data and re‐clustered them according to these data rather than the pathological dissection. We identified that FAM83D was highly expressed in IOC. To verify the role of FAM83D in the progression and metastasis, we used the isogenic ovarian cancer metastatic models, highly metastatic cells (HM) and non‐metastatic cells (NM). Overexpression of FAM83D significantly promoted cell proliferation, migration and spheroid formation. This was consistent with previous data showing that high FAM83D expression is associated with poor prognosis in cancer patients. Moreover, similar to the HM cells, the FAM83D‐overexpressing NM cells demonstrated stronger phosphorylation of the epidermal growth factor receptor (EGFR) and c‐Raf. This indicates that the action of FAM83D is mediated by the activation of the EGFR pathway. Taken together, this report suggested that FAM83D might be an excellent molecular marker to discriminate between IOC and LMP.

**Abbreviations**
AUC: area under the curve
CI: confidence interval
DSF: disease free survival
FFPE: formalin‐fixed, Paraffin‐embedded
GEO: gene expression omnibus
HGSOC: high‐grade serous ovarian carcinoma
HOSE: human ovarian surface epithelial
HR: hazard ratio
IHC: immunohistochemistry
IOC: invasive epithelial ovarian cancer
LGSOC: low‐grade serous ovarian carcinoma
LMP: low malignant potential ovarian tumour
OS: overall survival
PCA: principal component analysis
ROC: receiver operating characteristic
RT‐PCR: reverse transcriptase–polymerase chain reaction
TCGA: The Cancer Genome Atlas

## 1 INTRODUCTION

Low malignant potential tumour (LMP) is a semi‐malignant ovarian tumour, which was classified by the Federation of Gynecology and Obstetrics in 1961.^1^ LMP accounts for 15%‐20% of epithelial ovarian tumours.^2^ LMP is defined as a tumour with abnormal nuclear division and cell proliferation, lacking observable invasion into the stroma or invasion‐like implants.^3^ In contrast, invasive epithelial ovarian cancer (IOC), which represents approximately 70% of epithelial‐originated ovarian tumours, exhibits strong invasive properties. Based on the different invasiveness, the outcome of LMP and IOC differ considerably. The 5‐year survival rate of LMP patients is >90%, whereas that of IOC patients is <30%.^4^, ^5^ Therefore, the clinical management of patients with LMP and IOC is different. Considering the malignant status of the tumour and the desire for fertility‐sparing in patients, different operative procedures may be employed for LMP. In particular, preservation of fertility should be considered in younger patients.

Regarding the management of IOC, the gynecologist may recommend total hysterectomy and bilateral salpingo‐oophorectomy even in patients with Stage I ovarian cancer. In the extended resection, chemotherapy will be administered to eliminate invisible cancer cells, aiming to prevent relapse of ovarian cancer.^6^

Abdominal hysterectomy is the standard treatment for LMP. However, considering that the average age of LMP occurrence is 40 years, preservation of fertility may be important in these patients. In such cases, a more conservative surgical management—unilateral oophorectomy (ie removal of only one ovary)—may be considered. Since the managements of LMP and IOC are significantly different, accurate diagnosis of IOC and LMP is essential for the selection of the most appropriate treatment and will be beneficial to the patients. Indeed, approximately 20%‐30% of cases initially diagnosed with LMP are eventually confirmed to be IOC.^1^ The diagnosis is based on histopathological observation without the use of molecular markers, leading to inaccuracy in the diagnosis of LMP.^1^ Hence, the pathologist may often use terms such as ‘rule out LMP’ or ‘at least LMP’ in diagnostic reports.^7^, ^8^ The gene expression profile determines the phenotype of the tumour.^9^ Therefore, revealing the molecular differences between LMP and IOC and identifying useful molecular markers may increase the accuracy of diagnosis.

## 2 METHODS

### 2.1 Cell culture

The immortalized ovary surface epithelial cells (IOSE8) are cultured in M199/MCDB105 medium supplemented with 10% FBS and in 1% penicillin and streptomycin at 37°C in a humidified atmosphere of 5% CO2. The highly metastatic (HM) and non‐metastatic (NM) cells used in this study were isogenic cells lines derived from SKOV3.ip1 cells.^10^ The HM cells exhibited a strong metastatic signature, unlike NM cells which were shown to be non‐metastatic and failed to form detectable metastasis. Therefore, the HM/NM model offered a well‐controlled experimental system to study the metastasis of ovarian cancer. The cells are maintained in RPMI 1640 supplemented with 5% foetal bovine serum (Gibco, NY) and 1% penicillin and streptomycin at 37°C in a humidified atmosphere of 5% CO_2_. These two types of cells were kindly provided by Professor Alice ST Wong.

### 2.2 FAM83D‐overexpressing stable cell line

The FAM83D‐expressing plasmid was constructed by inserting the coding region sequence of FAM83D into the pcDNA3.1+ vector (Invitrogen, Burlington, Canada). To generate the FAM83D‐overexpressing stable cell line NM‐FAM83D, the FAM83D/pcDNA3.1 plasmid was transfected into NM cells using Lipofectamine 3000 (Invitrogen, Burlington, Canada). The NM cells transfected with an empty pcDNA3.1+ vector served as the control (NM‐Vector). Twenty‐four hours after transfection, G418 (150 μg/mL) was added for FAM83D stable expression cell line selection for 1 month. The expression of FAM83D was confirmed by real‐time polymerase chain reaction (PCR) and western blotting analysis.

### 2.3 Cell proliferation assay

The cell proliferation assay was performed using the IncuCyte ZOOMTM Live‐Cell Imaging and Analysis System according to the manufacturer's protocol. In brief, the NM‐Vector and NM‐FAM83D cells were seeded at a density of 3000 cells/well into 96‐well plate. The plate was maintained in the IncuCyte system for consecutive monitoring of cell proliferation. Images were recorded every 3 hours and cell confluency was analysed using the IncuCyte software (Essen BioScience; version 2018A).

### 2.4 Colony formation assay

The cells (500 cells) were seeded into a 100‐mm petri dish and incubated in a CO_2_ incubator at 37°C for 10 days or until cells in control plates formed colonies with substantially good size. Subsequently, the medium was removed and the colonies were stained with 0.5% crystal violate for 5 minutes and washed twice with phosphate buffered saline (PBS). The dishes were air‐dried at room temperature. Count images were captured and the number of colonies was counted using a stereomicroscope (Olympus, SZ61, Tokyo, Japan).

### 2.5 Western blotting

HM, NM, pCMV‐Vector and pCMV‐FAM83D cells were lysed using radioimmunoprecipitation assay (RIPA) buffer (20 mmol/L Tris‐HCl [pH 7.5], 150 mmol/L NaCl, 1 mmol/L Na_2_EDTA, 1 mmol/L EGTA, 1% NP‐40, 1% sodium, deoxycholate, 2.5 mmol/L sodium pyrophosphate, 1 mmol/L β‐glycerophosphate, 1 mmol/L Na_3_VO_4_, 1 µg/mL leupeptin and 1 mmol/L PMSF). Total proteins (20‐30 µg) were separated through polyacrylamide gel electrophoresis and transferred to a polyvinylidene difluoride membrane using a semi‐dry transfer system (Bio‐Rad). The membrane was incubated at 4°C overnight with specific primary antibodies for FAM83D (Biorbyt, orb183501), AKT (Cell Signal Technology, CST, MA, cat#: 4691), p‐AKT (CST, MA, cat#:4060), ERK1/2 (CST, MA, cat#:9102), p‐ERK1/2 (CST, MA, cat#: 9101), c‐Raf (CST, MA, cat#: 9422), p‐c‐Raf (CST, MA, cat#: 9421), EGFR (CST, MA, cat#: 4267), p‐EGFR (CST, MA, cat#:2234), P38 (CST, MA, cat#:9212), p‐P38(CST, MA, cat#:9211), N‐cadherin (CST, MA, cat#:13116), ZO1 (CST, MA, cat#:8193) and beta‐actin (CST, MA, cat#: 4970) used at a dilution of 1:1000. Subsequently, the membrane was incubated with secondary antibody (Bio‐Rad, CA, cat#: 1706515 in 1:5000 dilution) for 1 hour at room temperature. Clarity™ Western ECL Substrate (Bio‐Rad, cat#: 1705060) was used for the detection of protein signals. The signals were captured using the ChemiDoc™ XRS + Imaging Systems (Bio‐Rad, CA).

### 2.6 Wound healing assay

NM‐Vector and NM‐FAM83D cells were collected and washed once using Hank's buffer. Cells (1 × 10^5^) were seeded into a 12‐well plate and incubated until they reached >90% confluency. The samples were subsequently manually scratched using a P200 pipette. Images were acquired on days 0, 1, 2 and 3 using a light microscope (EVOS, ThermoFisher, MA, USA). The migration distance was measured using the ImageJ software.

### 2.7 Migration assay

To test the migration ability of cancer cells, NM‐Vector and NM‐FAM83D cells (2 × 10^4^ cells per well) were seeded to the upper chamber of a 24‐well Transwell plate (Corning, NY) containing serum‐free medium. The lower chambers contained the medium with 10% foetal bovine serum (Gibco NY). After 72 hours, the cells which remained on the upper surface were removed using a medical cotton swab. The cells at the lower surface and those which migrated to the bottom of the plates were fixed using 4% paraformaldehyde and stained with 0.5% crystal violate. Images were captured under a light microscope (EVOS, ThermoFisher, MA) with 40× magnification and the areas of staining were calculated using the ImageJ software.

### 2.8 Xenograft experiment

All standards and procedures of the animal experiments conducted in this study were approved by the Committee on the Ethics of Animal Experiments of the University of Macau (Protocol ID: UMARE‐029‐2017). Female, 4–6‐week‐old, NOD‐SCID mice were acquired from the Animal Facility, Faculty of Health and Sciences, University of Macau. Unless stated otherwise, the mice were fed ad libitum with standard rodent chow and water. For the xenograft experiment, the NM‐Vector or NM‐FAM83D cells (5 × 10^6^ cells in 200 µL Hank's buffer) were injected intraperitoneally. After 60 days, the mice were killed, the tumours were dissected and their number and weight were recorded.

### 2.9 Bioinformatics analysis

Gene transcriptional data were obtained from the National Center for Biotechnology Information Gene Expression Omnibus (GEO) including GSE9891, GSE12172, GSE27651, GSE14001, GSE57477, GSE36668, GSE30274 and GSE73551.

GSE12172 raw data were normalized in MultiExperiment Viewer (MeV). The differential gene expressions (fold changes >2, *P* < 0.001) were identified using the ‘Linear Models for Microarray’ method (LIMMA, http://mev.tm4.org).^11^ The gene copy number variation of FAM83D and FAM81B was obtained from the Oncomine online database.^12^ The correlation of gene expression with tumour stage and tumour grade were evaluated using the Ovarian cancer database of the Cancer Science Institute of Singapore and statistical significance was calculated using the Mann‐Whitney test. For the analysis of the percentage of overall survival (OS) and disease‐free survival (DFS), the log‐rank test was used to compare the survival expectation of a group with different gene expression.^13^

### 2.10 Human sample PCR and ethics

Six human samples with a definitive diagnosis of high‐grade serous ovarian carcinoma were obtained from the Second Affiliated Hospital of Harbin Medical University (Harbin, China). Total RNA was extracted using a formalin‐fixed paraffin‐embedded (FFPE) sample processing kit according to the manufacturer's protocol (Quantigene, Miami). cDNA was synthesized using the SuperScript™ IV First‐Strand Synthesis kit (Invitrogen). A standard PCR reaction was performed using the following primers: forward primer, GCCTTCTACCAGGGCGCCTAC; reverse primer, ACGTCCATGACCACTGCAATCAC. All patients were informed regarding the purpose of the research and provided informed consent. These experiments were approved by the Ethics Committee of Harbin Medical University.

### 2.11 Immunohistochemistry

All clinical samples were analysed by standard immunohistochemical staining at the same time. Briefly, 5‐μm sections were deparaffnized, rehydrated and heat‐antigen retrieved. After incubated in 3% H_2_O_2_ for 15 minutes, samples were incubated with goat serum for 1 hour at room temperature. The sections were incubated with FAM83D primary antibody (Biorbyt, cat#:orb183501, 1:200) at 4°C overnight, and followed by incubation with the secondary antibody (MaxVision TM HRP‐Polymer anti‐Mouse/Rabbit IHC Kit, Maxim biotech) according to the manufactory protocol. The peroxidase reaction was then detected with DAB for 5 minutes. The grading criteria were as follows: signalling strength (negative 0, weak 1, moderate 2 and strong 3). Immunohistochemistry (IHC) scores were calculated as the sum of location scores and signalling strength scores.

### 2.12 Statistical analysis

Data are presented as means ± standard error of mean (SEM) from at least three independent experiments. Student's *t* test (with Welch's correction) was performed for comparison between two groups and *P* < 0.05 denoted statistical significance. A receiver operating characteristic curve (ROC) was generated to estimate the diagnostic ability of a parameter. Principal component analysis (PCA) was employed to distinguish the IOC from the LMP samples (Gastinel, 2012). PCA based on transcriptomic data was used to distinguish patients.

## 3 RESULTS

### 3.1 Re‐clustering of LMP and IOC cases based on the transcriptomic data

**Figure 1:**
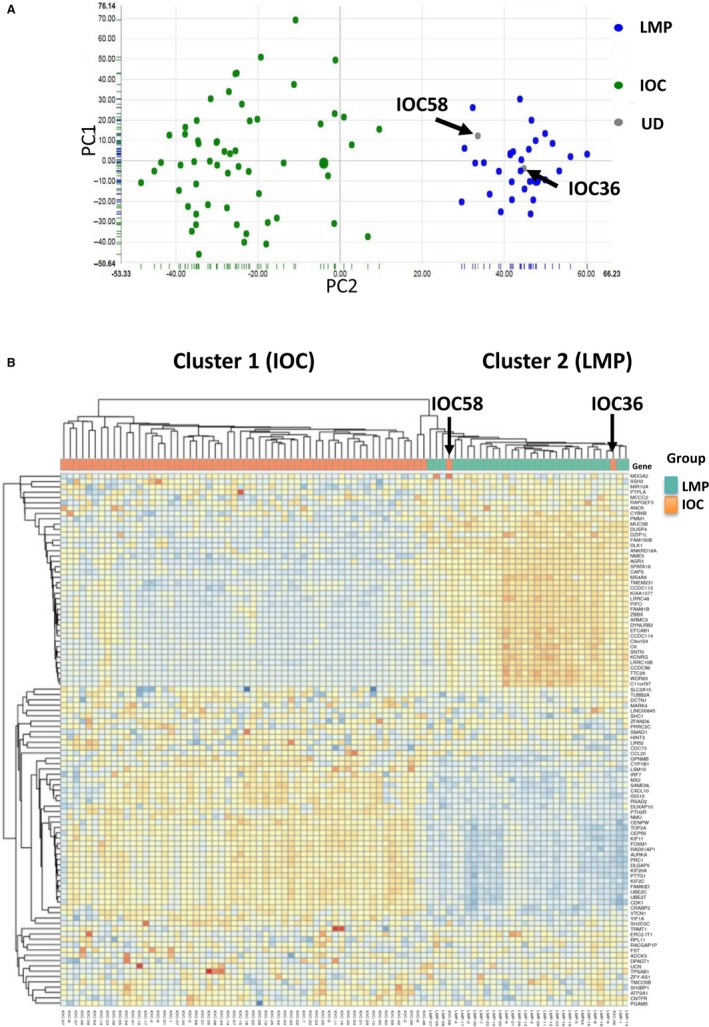
Using transcriptomic data to accurately identify IOC and LMP. (A) The clinically/pathologically defined IOC and LMP, as well as patient transcriptomic data were reanalysed using PCA analysis. (B) Hierarchical clustering was employed to classify the clinically/pathologically defined IOC and LMP. The dataset used in this figure was GSE12172, which contained 60 IOC and 30 LMP samples. IOC, invasive epithelial ovarian cancer; LMP, low malignant potential ovarian tumour; PCA, principal component analysis

To understand the differences between the molecular profiles of IOC and LMP, we initially classified the patients with LMP or IOC under a definitive pathological diagnosis. The GSE12172 dataset including 30 LMP and 60 malignant IOC cases was used to establish a training methodology for the accurate differentiation of IOC and LMP. We re‐clustered the cases using whole transcriptomic data from both pathologically defined LMP and IOC cases. The results of the PCA analysis using whole transcriptomic data suggested that the LMP and IOC could be accurately separated except for two cases (IOC36 and IOC58). These two cases were originally classified as IOC according to the pathological diagnosis. However, after re‐clustering, they were clustered into the LMP group (Figure 1A). In addition, hierarchical clustering data analysis was employed to re‐cluster the cases (Figure 1B). Similar to the first analysis, IOC36 and IOC58 were identified as LMP rather than IOC. Therefore, these two cases were probably rare misdiagnosed cases. To further identify differences between the molecular expression of LMP and IOC, we excluded these two diagnostically inconsistent cases. Comparison of the newly defined groups showed that 409 and 593 genes had higher and lower expression, respectively in the IOC group versus the LMP group. The eight genes with the most significant differential expression were further analysed in the survival analysis to evaluate the association between expression and overall patient survival. Among the highly expressed genes in the IOC group, FAM83D and SH2D3C showed the strongest association with poor survival (hazard ratio [HR] 1.720; 95% confidence interval [CI] 1.290‐2.294 and HR 1.287; 95% CI 0.985‐1.683, respectively). Among the genes with lower expression in the IOC group, FAM81B and AGR3 demonstrated the strongest correlation with better survival (HR 0.492; 95% CI 0.183‐1.321 and HR 1.287; 95% CI 0.419‐0.753, respectively) (Table 1). The Kaplan–Meier survival curves and the median survival times of the tested genes are shown in Figure S1 and Table 1. The molecular detection of genes with higher expression is easier. Therefore, we propose FAM83D as a potential molecular marker to distinguish IOC from LMP.

**Table 1:** The hazard ratio (HR) on survival of the most significantly differential expressed gene in IOC vs LMP

Gene	Gene expression different in IOC vs LMP [Log (Fold Change)]	‐Log (*P* value)	HR (95% CL)	Median Survival Time of low group vs high group (mo)
FAM83D	3.0237	61.4442	1.720 (1.290‐2.294)***	66.57 vs 43.00
SH2D3C	3.2686	30.3251	1.287 (0.985‐1.683)	57.00 vs 36.27
VTCN1	3.0068	25.6068	0.914 (0.765‐1.094)	48.06 vs 49.73
SGCA	3.4404	64.923	0.961 (0.803‐1.150)	41.60 vs 45.53
SNTN	−4.8272	64.923	0.842 (0.632‐1.122)	50.03 vs 57.00
FAM81B	−4.3291	54.622	0.492 (0.183‐1.321)	54.83 vs N.A.
SSH2	−4.5027	47.9674	1.029 (0.786‐1.347)	43.93 vs 47.17
AGR3	−4.1836	39.0844	0.561 (0.419‐0.753)	41.87 vs 77.33

Abbreviations: IOC, invasive epithelial ovarian cancer; LMP, low malignant potential. N.A. is the median survival time for high expression group for FAM81B is not available. ***P < 0.0001.

### 3.2 FAM83D participates in the migration of ovarian cancer

Since the expression FAM83D was shown to be strongly associated with the overall patient survival, we hypothesized that FAM83D may promote the progression and metastasis of ovarian cancer. Here, we used the isogenic ovarian cancer metastatic models—highly metastatic (HM) cells and non‐metastatic (NM) cells—to test this hypothesis. It is worth noting that the use of the HM and NM cells in this project is just the tools to verify the role of FAM83D in cancer metastasis, but not to mimic the different between IOC and LMP.

**Figure 2:**
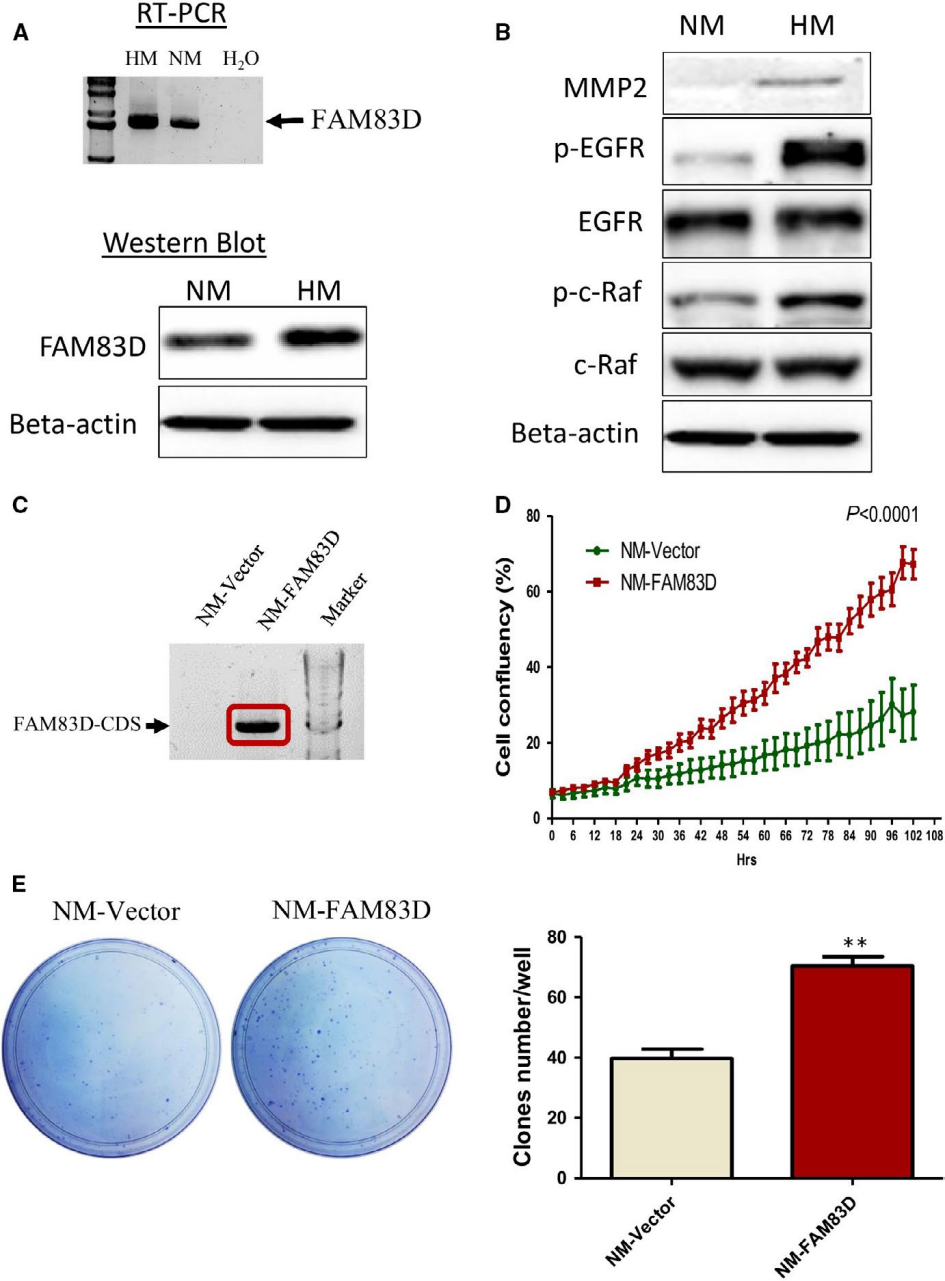
High expression of FAM83D in highly metastatic cells and the role of FAM83D in the proliferation of ovarian cancer cells. (A) Detection of FAM83D mRNA and protein levels from HM and NM cells using reverse‐transcript PCR (RT‐PCR) and Western blotting. (B) Western blotting analysis of the MMP2 and EGFR‐related pathway. (C) RT‐PCR showed the high expression of FAM83D in the stable cell line NM‐FAM83D. The FAM83D/pcDNA‐ or pcDNA (control vector)‐transfected cells were transferred into NM cells and used G418 for selection for one month. (D) The proliferation of NM cells after overexpression of FAM83D was measured using the IncuCyte^®^ S3 Live‐Cell Analysis System for 102 h. (E) The ability for single cancer cell tumorigenesis was evaluated through a colony formation assay. The data are presented as means ± SEM; **P < 0.01 against the NM‐vector control. HM, highly metastatic; NM, non‐metastatic; RT‐PCR, reverse transcriptase–polymerase chain reaction; MMP2, matrix metalloproteinase‐2; EGFR, epidermal growth factor receptor; SEM, standard error of mean

Western blotting and RT‐PCR analysis showed that the expression of FAM83D was higher in HM cells compared with that observed in NM cells (Figure 2A). In addition, we tested the expression of metastatic marker genes and the activation of the EGFR pathway. The results revealed high expression of matrix metalloproteinase‐2 (MMP2) and increase in protein phosphorylation of EGFR and c‐Raf in HM cells (expressing high levels of FAM83D) (Figure 2B). Our results are consistent with those of previous studies showing that HM cells exhibit strong metastatic properties.^10^ Regarding the high expression of FAM83D observed in HM cells, current data also suggest that FAM83D may be involved in the malignant characteristics of HM cells.

To further investigate the role of FAM83D in ovarian cancer cells, we established a stable cell line overexpressing FAM73D in NM cells (NM‐FAM83D; Figure 2C). The cell line with an empty vector served as the mock control (NM‐Vector). Our results showed that overexpression of FAM83D (NM‐FAM83D) significantly promoted cell proliferation compared with the NM‐vector (Figure 2D). Moreover, the colony formation assay suggested that overexpression of FAM83D can increase the colony number and enhance the colony growth of ovarian cancer cells (Figure 2E).

**Figure 3:**
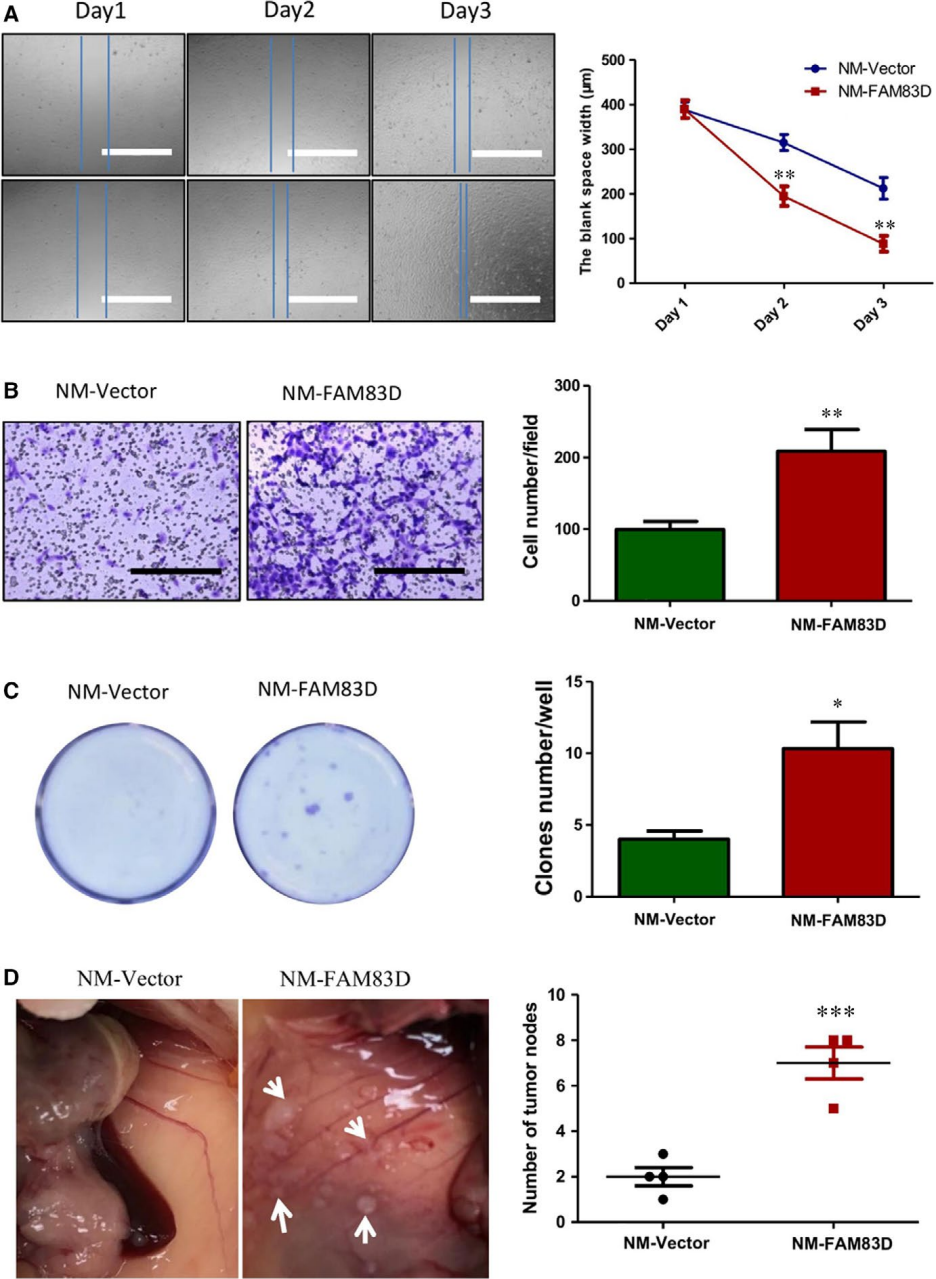
Overexpression of FAM83D enhanced the migration and tumour formation in ovarian cancer. (A) The motility of ovarian cancer cells was significantly increased after overexpression of FAM83D. The motility of NM‐FAM83D and NM‐Vector was tested using a wound healing assay. Scale bar = 1 mm. (B) Cell migration was evaluated using the Transwell assay. The cells that migrated through the membrane were stained using 0.5% crystal violate. Scale bar = 1 mm. (C) In the same Transwell assay, the cells that migrated through the membrane and clones formed in the plate were stained using crystal violet. (D) The dissemination of NM cells after overexpression of FAM83D was evaluated in NOD‐SCID mice through intraperitoneal injection. The tumours in the mice were dissected and counted. The data are presented as means ± SEM; * *P* < 0.05, **P < 0.01 against the NM‐vector control. NM, non‐metastatic; SEM, standard error of mean

In both wound healing and Transwell assays, the increase in FAM83D significantly enhanced cell motility (Figure 3A) and promoting migration of cancer cells in the Transwell chambers (Figure 3B,C). To further verify the role of FAM83D in the peritoneal dissemination of ovarian cancer, we intraperitoneally injected NM‐FAM83D and NM‐vector cells into NOD‐SCID mice. In this xenograft model, the number of tumours in the NM‐FAM83D group was significantly higher than that reported in the NM‐vector group. The results clearly suggested that FAM83D‐overexpressing cells possess stronger invasive ability in the peritoneal cavity (Figure 3D).

### 3.3 FAM83D is up‐regulated in ovarian carcinoma and correlated with ovarian malignant characteristics

**Figure 4:**
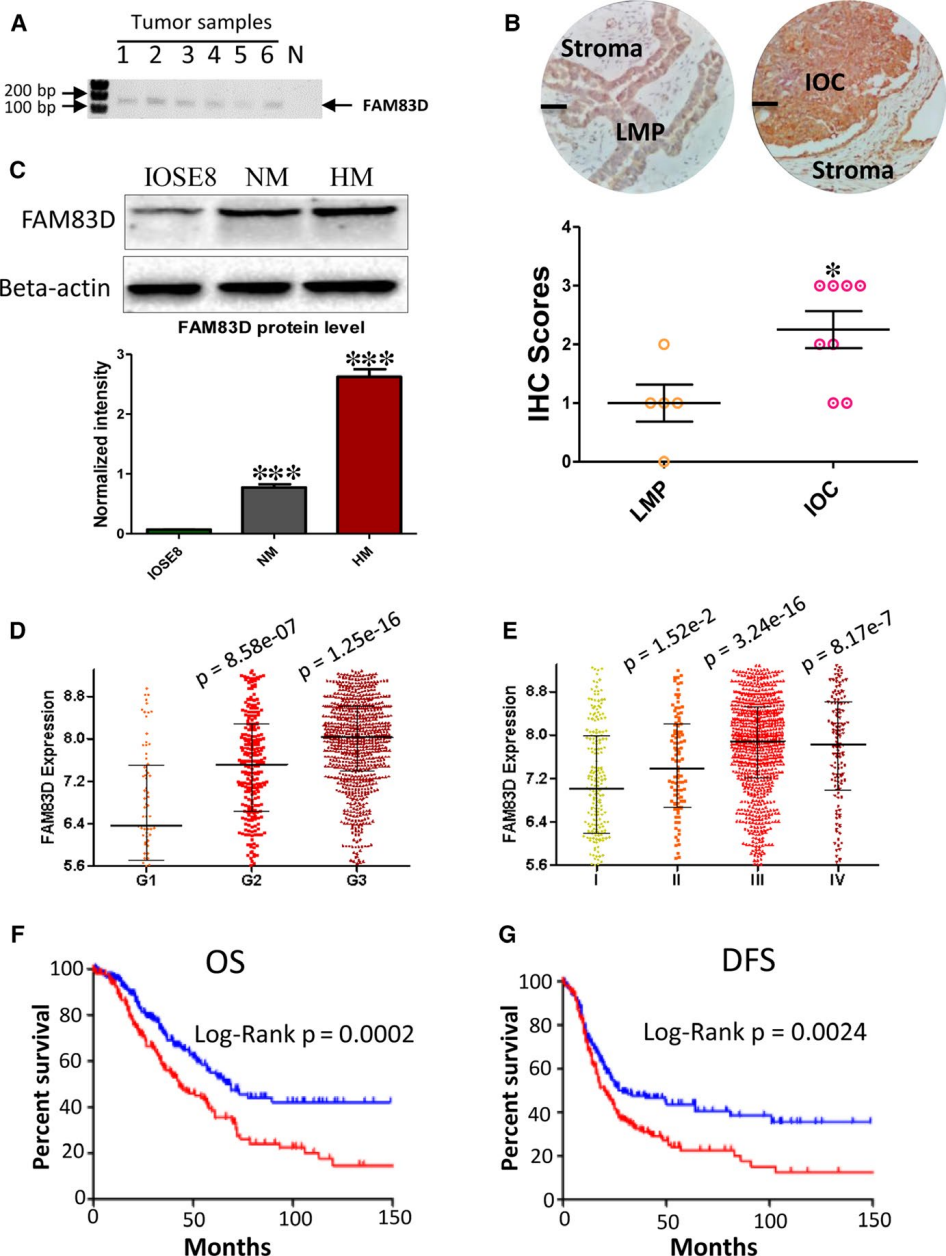
Strong FAM83D expression in IOC patient samples and high expression of FMA83D are positively associated with prognosis. (A) RT‐PCR was performed to detect the mRNA expression of FAM83D in formalin‐fixed paraffin‐embedded tumour tissues. (B) IHC staining of FAM83D in IOC and LMP samples. Upper: The representative staining of FAM83D in IOC and LMP samples (Scale bar = 50 μm). Lower: The IHC scores of IOC and LMP samples IOC (n = 8) and LMP (n = 5) (*P = 0.024). (C) The protein levels of FAM83D in immortalized ovary cell (IOSE8) and ovarian cancer cells (NM and HM) were detected using Western blotting. (D) The expression level of FAM83D in different grades of tumour tissue. The Mann‐Whitney test was performed to test other groups against the grade 1 group. (E) The expression level of FAM83D in different stages of metastasis in tumour tissues. The Mann‐Whitney test was performed to test other groups against the stage 1 group. (F) The overall survival curve of patients with high expression of FAM83D (upper 25%, n = 202) and low expression of FAM83D (lower 25%, n = 203). (G) The disease‐free survival curve of a patient with high expression of FAM83D (upper 25%, n = 176) and low expression of FAm83D (lower 25%, n = 177). The log‐rank test was performed to compare patient survival curves between two groups. IOC, invasive epithelial ovarian cancer; RT‐PCR, reverse transcriptase–polymerase chain reaction

To verify the expression of FAM83D in patients with high‐grade serous ovarian cancer, we measured the expression of FAM83D using RT‐PCR. Our data showed that all six samples investigated demonstrated significant expression of FAM83D (Figure 4A). IHC was performed to investigate the expression of FAM83D in the IOC and LMP FFPE (Formalin‐fixed, Paraffin‐embedded) samples. The results agreed with whole transcriptomic data that a significant higher of FMA83D immunoreactivities was found in IOC samples when compared with LMP (IOC n = 8; LMP n = 5) (Figure 4B). Furthermore, we investigated whether the aberrant expression of FAM83D was an oncogenic event. We compared the expression of FAM83D in immortalized ovary epithelial cell (IOSE8) and ovarian cancer cells (NM and HM). The protein level of FAM83D was significantly lower in IOSE8 cells compared with that observed in HM and NM cells (Figure 4C). Since high expression may be due to a change in gene copy number, we extracted the FAM83D gene copy number data in patients with ovarian cancer from The Cancer Genome Atlas (TCGA) database (Figure S2A). We found that the gene copy number of FAM83D in IOC was significantly higher than that observed in healthy ovary tissues. These data indicate that an increase in the copy number may contribute to the up‐regulation of FAM83D in some patients. To investigate whether the expression of FAM83D was clinically relevant, we analysed its association with the malignant characteristics of ovarian cancer. Our data suggest that FAM83D is positively correlated with ovarian cancer pathological grades and stages (Figure 4D,E). Furthermore, high FAM83D expression was associated with poor prognosis (OS and DFS time) in patients. The median OS times in the FAM83D high‐ and low‐expression groups were 43.00 and 66.57 months, respectively (Figure 4F). The median DFS time in the FAM83D high‐ and low‐expression groups were 20.00 months and 27.17 months, respectively (Figure 4G). These clinical data are consistent with our hypothesis that the FAM83D is involved in ovarian cancer metastasis and hence, the high expression of FAM83D is potentially a good indicator of IOC.

### 3.4 Up‐regulation FAM83D triggers the EGFR signalling pathway and promotes migration and proliferation of cancer cells

**Figure 5:**
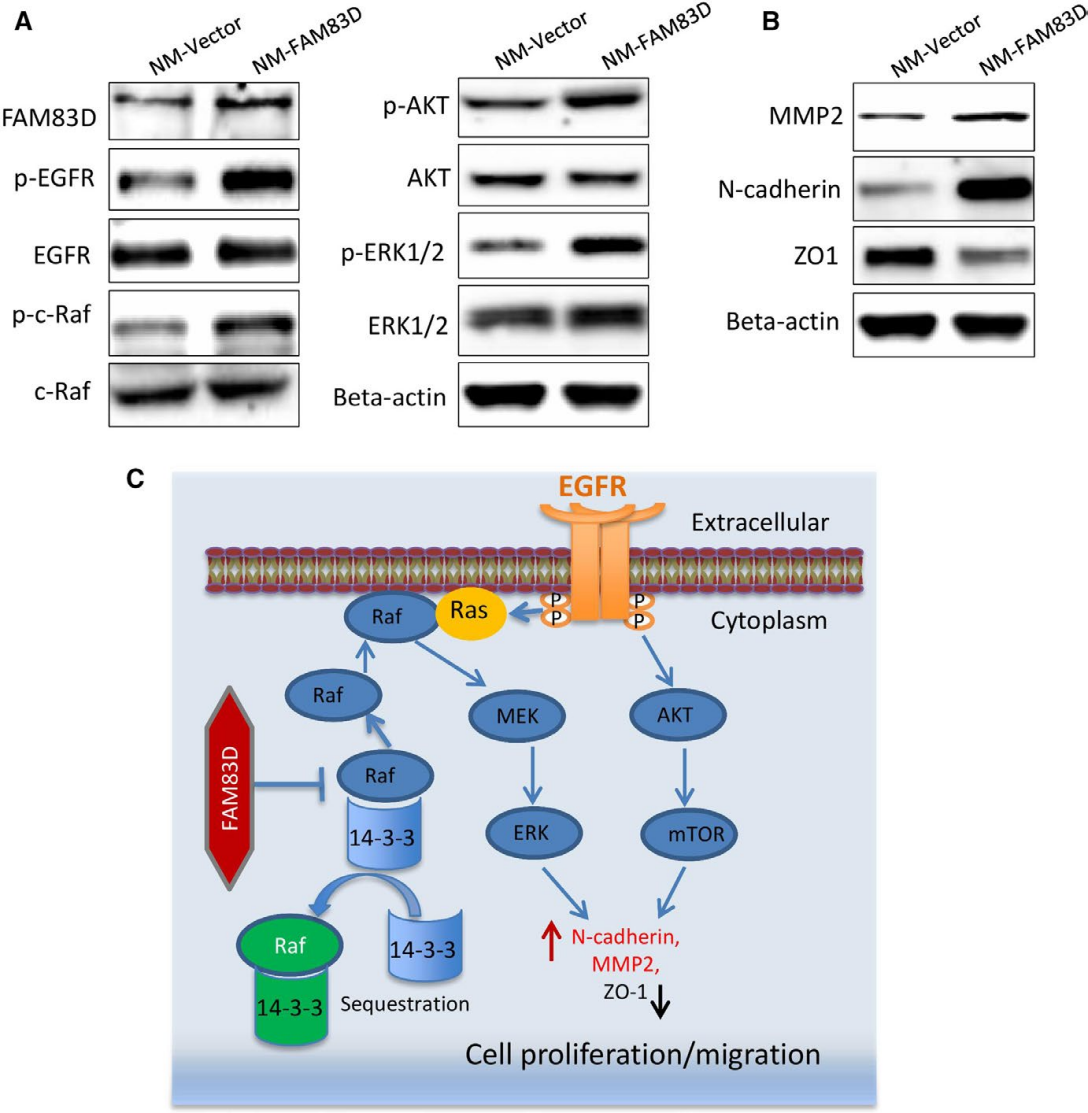
FAM83D promotes activation of the oncogenic pathways. (A) The protein levels of FAM83D, EGFR, p‐EGFR and c‐Raf, p‐c‐Raf, ERK1/2, p‐ERK1/2, AKT and p‐AKT in the NM‐vector and NM‐FAM83D cells were quantified using Western blotting. (B) The protein levels of MMP2, N‐cadherin and ZO1 in the NM‐vector and NM‐FAM83D cells were quantified using Western blotting. (C) Schematic diagram summarizing the molecular mechanism through which FAM83D enhanced the proliferation and migration of ovarian cancer cells. EGFR, epidermal growth factor receptor, NM, non‐metastatic; MMP2, matrix metalloproteinase‐2

A previous study reported that the FAM83 protein family has a highly conserved N‐terminal domain of unknown function (DUF1699) that able to prevent the interaction between cRaf and the regulatory 14‐3‐3 protein. Since the regulatory 14‐3‐3 protein could induces the sequestration of c‐Raf and reduce the recruitment of c‐Raf to plasma membrane, FAM83D therefore could enhance the membrane localization of cRaf. The membrane localization of c‐Raf can be activated by the EGFR/Ras signalling pathway, resulting in the malignant transformation of human epithelial cells.^14^ Therefore, we proposed that the overexpression FAM83D might promote the migration and proliferation of cancer cells through activates the EGFR and c‐Raf signalling pathways. To validate this hypothesis, we further tested the phosphorylation of EGFR/c‐Raf, as well as their downstream signalling proteins AKT and ERK1/2 using the FAM83D‐overexpressing cell lines. Our results showed that the overexpression of FAM83D significantly increased the phosphorylation of all the molecules in NM‐FAM83D cells versus the control NM‐Vector (Figure 5A). Since the activation of p38 MAPK pathway may also resulted in cell proliferation in cancer cells, we also tested P38 MAPK phosphorylation. But the result suggested the overexpression of FAM83D does not affect this pathway (Figure S2B). This indicates FAM83D selectively affecting the cell signalling in ovarian cancer cells. Since activation of the EGFR/c‐Raf pathway may directly enhance epithelial‐mesenchymal transition (EMT), we further measured the levels of the following EMT markers: MMP2, N‐cadherin and ZO1 (Figure 5B). Consistent with the signalling analyses, the overexpression of FAM83D markedly increased the levels of N‐cadherin and MMP2, while reducing the expression of ZO1. In summary, our data demonstrated that the up‐regulation of FAM83D was involved in the progression of ovarian cancer and induced oncogenic events through activation of the EGFR pathway, promoted c‐Raf membrane localization and activated EGFR‐related downstream signal pathways which resulted in the proliferation and migration of cancer cells (Figure 5C).

### 3.5 FAM83D was effective in the diagnosis of LMP and IOC

**Figure 6:**
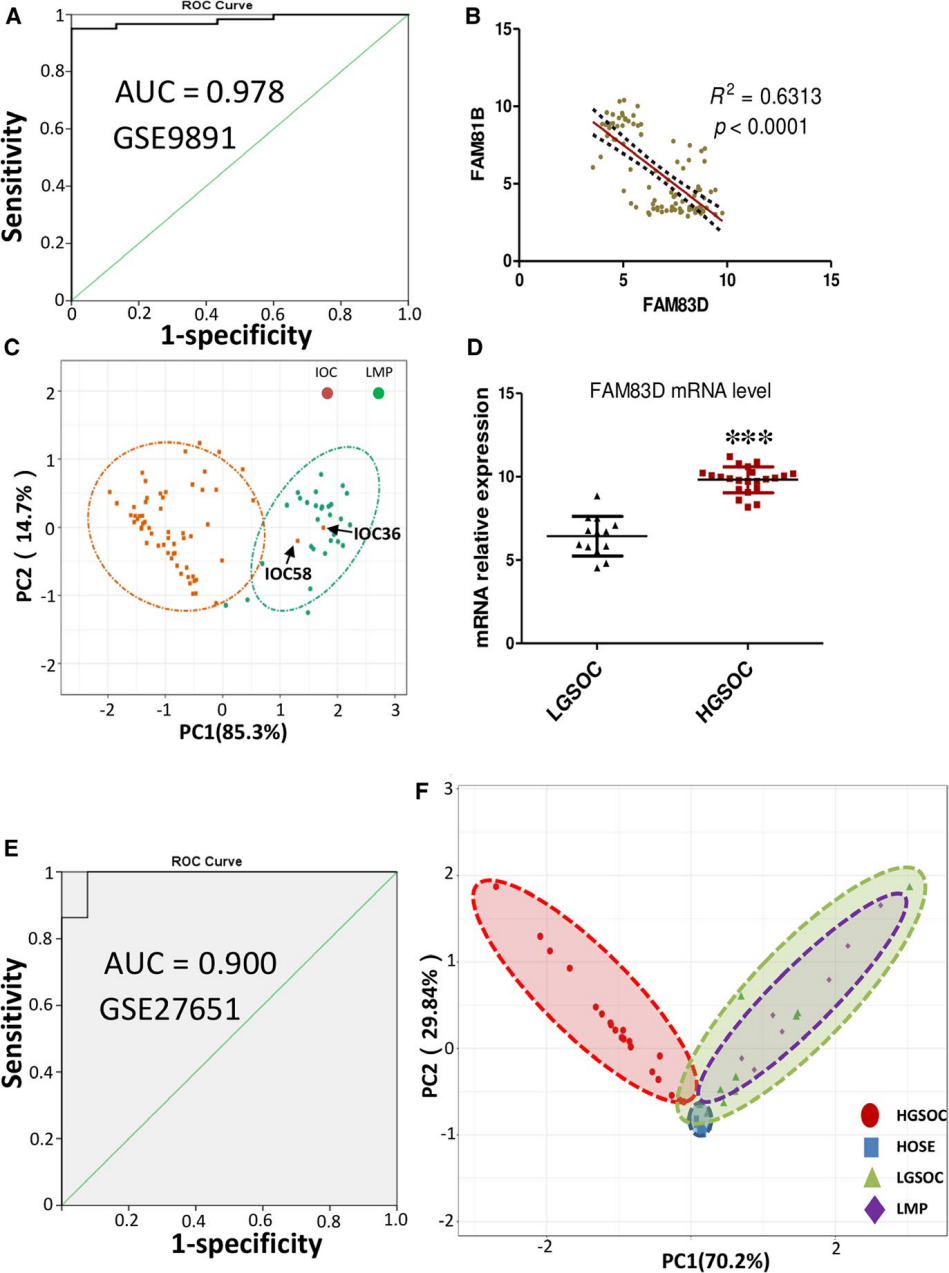
FAM83D was effective in distinguishing IOC from LMP and identifying LGSOC. (A) ROC plot of FAM83D expression in the diagnosis of IOC and LMP. (B) The correlation between the expression of FAM83D and FAM81B through Pearson correlation analysis. (C) PCA analysis performed regarding the diagnosis of IOC and LMP by FAM83D and FAM81B. (D) The mRNA levels of FAM83D in LGSOC and HGSOC tumours were extracted from the online dataset GSE27651. (E) ROC plots of FAM83D expression in the diagnosis of HGSOC and LGSOC. (F) PCA analysis performed regarding the discrimination of HOSE, LMP, LGSOC and HGSOC tissues by FAM83D and FAM81B. IOC, invasive epithelial ovarian cancer; LMP, low malignant potential ovarian tumour; LGSOC, low‐grade serous ovarian carcinoma; ROC, receiver operating characteristic; PCA, principal component analysis; HGSOC, high‐grade serous ovarian carcinoma; HOSE, human ovarian surface epithelial

The ROC is a methodology to illustrate the ability of a parameter to accurately diagnose a disease. The area under the curve (AUC) of the ROC curve is an index evaluating the performance of a parameter in the discrimination of certain diseases.^15^ To test the potential of FAM83D in the diagnosis of LMP and IOC in clinical practice, the dataset GSE9891 was selected as a testing model. The results showed that FAM83D could distinguish IOC from LMP with an AUC of 0.978 (Figure 6A). We collected other GEO datasets and analysed the capacity of FAM83D in the diagnosis of LMP and IOC using a ROC curve. The results are summarized in Table 2. Within the tested datasets, the lowest AUC was 0.742 and the highest up to 1.0. In most cases, the AUC was close to or higher than 0.9, which strongly implies that FAM83D could serve as a marker to distinguish IOC from LMP. In this analysis, we also found that the expression of FAM81B was mutually exclusive with that of FAM83D in both IOC and LMP (Figure 6B). We suggest that the use of these two genes as markers may further increase diagnostic accuracy for LMP and IOC by replacing transcriptome‐wide PCA analysis. The results showed that FAM83D and FAM81B could identify IOC36 and IOC58 as LMP (Figure 6C), which is consistent with the analysis using whole transcriptome data (Figure 1A). Therefore, detecting the expression of these two genes may provide a molecular diagnosis approach to identifying LMP and IOC with significantly improved accuracy compared with that offered by histopathological observation.

**Table 2:** The ROC analysis based on FAM83D was performed on diagnosis IOC, HGSOC, LGSOC and LMP

GEO accession	Country	IOC	HGSOC	LMP	LGSOC	AUC
GSE9891	Australia	267	/	18	/	0.978
GSE12172	USA	60	/	30	/	0.981
GSE27651	USA	/	21	8	/	0.900
GSE27651	USA	/	21	/	13	0.989
GSE14001	USA	/	12	/	10	0.933
GSE57477	Sweden	66	/	6	/	0.742
GSE36668	Norway	4	/	4	/	1.0
GSE30274	Japan	26	/	5	/	0.892
GSE73551	UK	/	13	/	7	0.879

Abbreviations: AUC, Area under curve; HGSOC, High‐grade serous ovarian carcinoma; IOC, invasive epithelial ovarian cancer; LGSOC, Low‐grade serous ovarian carcinoma; LMP, low malignant potential.

IOC is a general term for ovarian cancer including many histotypes, such as serous, endometrioid, clear and mucinous carcinoma. Serous ovarian carcinoma is the most common subtype classified into two subtypes according to the World Health Organization (WHO): high‐grade serous ovarian carcinoma (HGSOC) and low‐grade serous ovarian carcinoma (LGSOC). These two types of serous ovarian carcinoma are distinct diseases according to their progression, chemo‐sensitivity and prognosis. Of note, LGSOC is associated with a younger age at diagnosis (median age: 45‐57 years). The properties of LGSOC, including slower growth and resistance to chemotherapy, are similar with those of LMP. Data analysis also showed that the expression of FAM83D was significantly lower in LGSOC compared with that observed in HGSOC (Figure 6D). The AUC of FAM83D in the diagnosis of HGSOC and LGSOC was >0.8 (Figure 6E and Table 2), suggesting that FAM83D may also be a potential marker to distinguish HGSOC from LGSOC. To verify this hypothesis, we compared the gene expression profiles from GSE27651 dataset, which contains data for HGSOC, LGSOC, LMP and human ovarian surface epithelial (HOSE) cancers. The clustering clearly indicated that the gene expression profile of HGSOC was significantly different to that of others. Moreover, the LGSOC pattern was similar to that of LMP (Figure 6F). Therefore, utilization of FAM83D may effectively discriminate HGSOC from LGSOC (Table 2).

## 4 DISCUSSION

Ovarian cancer is the most lethal type of gynecological cancer, causing 151 000 deaths worldwide annually.^10^, ^16^ LMP is defined according to the morphological structure of tumour tissue under the microscope, due to the atypical invasion of the stroma.^17^ Although most of LMP will maintain a mild status for a long time, a small proportion of LMP possesses the potential to progress into invasive ovarian cancer. The notion that LMP is the early stage of IOC or that LMP is only one type of neoplasm which is completely different from IOC remains controversial. We prospected that the molecular profiles of IOC and LMP are different and unique. However, currently, there is no specific and highly sensitive biomarker for distinguishing IOC from LMP. This lack of accurate markers leads to incorrect diagnoses of IOC or LMP and inappropriate treatment, leading to adverse consequences. In this study, we uncovered the differences between the molecular profiles IOC and LMP using whole transcriptome data. Using functional analysis, we suggested that FAM83D was a reliable molecular marker that could improve the diagnosis of IOC and LMP.

Whole transcriptomic profiling analysis is useful for the diagnosis of tumours and molecular classification.^18^ The transcriptomic‐confirmed cases were used for comparison of the transcriptome profiles and differentially expressed genes were identified. Using a similar approach, in this study we identified the FAM83D and FAM81B genes which can distinguish IOC from LMP as the whole transcriptome perform. Due to FAM81B is down‐regulated, whereas and FAM83D is highly expressed in IOC. Considering that FAM83D is a highly expressed gene, it may be a better biomarker for the diagnosis of IOC and LMP than FAM81B. The high expression renders these markers easy for detection through IHC—a traditional ‘golden standard method’ in clinical pathology diagnosis.

Tracking of a molecular signature in different types of ovarian tumours is essential to accurately distinguish different tumours and select the most appropriate management protocol for patients. Currently, LMP is considered to be an intermediate status between benign and ovarian cancer. In this study, we also demonstrated that using FAM83D and FAM81B may further enhance the AUC of the diagnosis. Although the LGSOC and HGSOC are considered to originate from the fallopian tube,^19^ their properties and clinical outcomes are very different. When compared with HGSOC, LGSOC is a rare (account for 10% of serous ovarian cancer cases), slow‐growing cancer and generally more resistant to cytotoxic chemotherapy. Although most patients with LGSOC are diagnosed with advanced disease, they are associated with prolonged survival.^20^ Our results suggest that LMP and LGSOC share similar gene expression profiles. This finding is consistent with data from Bonome et al, showing that the gene expression profile of LGSOC is similar to that of LMP, but different from that of HGSOC.^21^ In 2014, the WHO proposed that ovarian tumours can be classified into two categories based on their clinicopathological and molecular features. In that report, they divided LGSOC into type I tumour and its precursor is LMP cell. In contrast, the HGSOC was classified as type II tumour.^19^ In this study, we propose that FAM83D is a good method for the accurate diagnosis of IOC and LMP, and differentiation of HGSOC from LGSOC.

There are several studies showing FAM83D gain of function in several types of cancer,^22^, ^23^ especially breast cancer.^22^, ^24^ Interestingly, up‐regulation of the FAM83D predicts cancer patients (breast, lung, liver) with a high risk of mortality.^22^ Wang Z et al reported that FAM83D promotes the proliferation of cancer cells through inhibition of the tumour suppressor FBXW7 in breast cancer. FAM83D gain of function activated the PI3K/mTOR signalling pathway and resulted in cell division in breast cancer.^24^ Dong et al investigated the role of FAM83D in liver cancer, showing that FAM83D contributes to the proliferation of cancer cell and colony formation through activation of the MEK/ERK pathway.^26^ In this study, using the isogenic cell model, we suggested that high expression of FAM83D significantly promotes proliferation, migration and spheroid formation in ovarian cancer cells (Figures 2 and 3). Even the NM cells are ovarian cancer cells that cannot represent the LMP, this isogenic cell model still suggesting the above functional changes are caused by the increase in the expression of FAM83D, which stimulates the c‐Raf/MEK/ERK1/2 pathway (Figure 5). This evidence confirmed that the role of FAM83D is similar to that observed in phenotypes of other cancers. The DUF1669 domain of the FAM83 proteins, which is demonstrated participated in inhibit the regulatory 14‐3‐3 proteins to sequestrate c‐Raf. Hence, FAM83D may increase the membrane recruitment of c‐Raf and further active the MEK/ERK pathways.^25^ Interestingly, we have demonstrated FAM83D overexpression not only activate the MEK/ERK pathway, but also activation of EGFR and PI3K/AKT pathway (Figure 5A). We speculate that the FAM83D initiates MEK/ERK1/2 could up‐regulate the expression of MMP2 expression, which active EGFR in a ligand‐dependent mechanism. Indeed, previous reports also suggested the knockdown of FAM83D with shRNA will increase the activation of EGFR/MAPK pathway.^25^ The overall signalling pathway is summarized in Figure 5C.

In conclusion, we demonstrated that FAM83D is a highly expressed gene in IOC and correlated with tumour stage and grade. Functional analyses further suggested the role of FAM83D in promoting the proliferation, migration and metastasis of ovarian cancer cells. Therefore, FAM83D may be an excellent marker for distinguishing IOC from LMP and may contribute to differentiating HGSOC from LGSOC.

## CONFLICT OF INTEREST

The authors have no competing interests to disclose.

## AUTHOR'S CONTRIBUTIONS

Conception and design: Qingyu Zhang, Yufei Jiao, Leo TO Lee; Development of methodology: Qingyu Zhang, Shan Yu, Alice Sze Tsai Wong; Acquisition of data: Qingyu Zhang, Samson Ian Sam Lok; Analysis and interpretation of data: Qingyu Zhang, Shan Yu, Yufei Jiao, Leo TO Lee.
