# Supplementary material for: FAM83D promotes ovarian cancer progression and its potential application in diagnosis of invasive ovarian cancer
Source: J Cell Mol Med. 2019 Apr 30;23(7):4569–81. doi: 10.1111/jcmm.14360 (PMC6584551; doi:10.1111/jcmm.14360)
Supplement: Supplementary file 1 [file JCMM-23-4569-s001.pdf]

Supplementary Figure 1

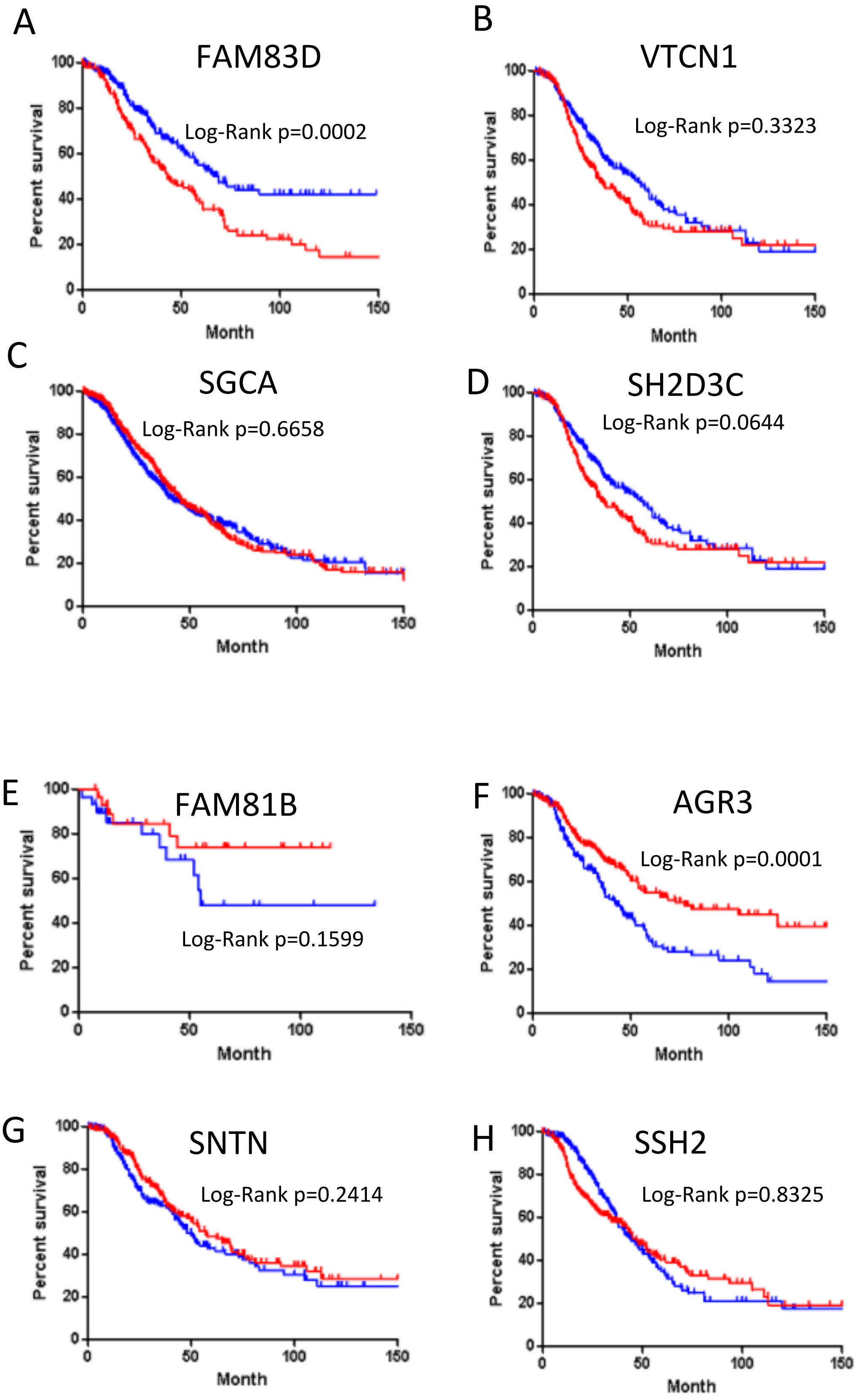

Fig. S1. Kaplan–Meier survival curves: Expression status of the investigated genes in patients with ovarian cancer.

Supplementary Figure 2

A

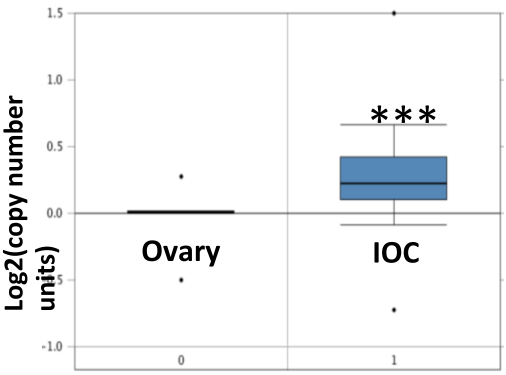

B

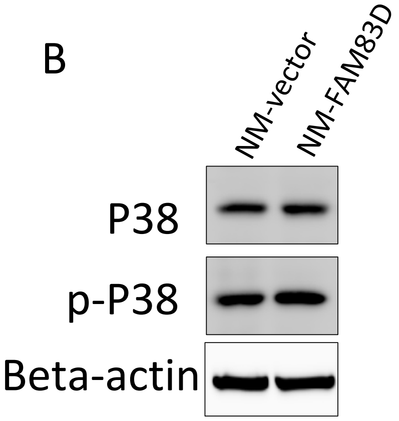

Fig.S2. A. Alterations in the gene copy number in IOC was evaluated using data from the Oncomine online database (https://www.oncomine.org). **B**. The protein levels of P38 and p-P38 in the NM-vector and NM-FAM83D cells were quantified using western blotting.
